# The inverse association between cancer and Alzheimer’s disease: evidence from neuropathological studies

**DOI:** 10.1002/alz.087469

**Published:** 2025-01-09

**Authors:** Erin L. Abner, Shama D Karanth, Khine Z Aung, Peter T Nelson

**Affiliations:** ^1^ College of Public Health, University of Kentucky, Lexington, KY USA; ^2^ University of Kentucky, Lexington, KY USA; ^3^ Sanders‐Brown Center on Aging, Lexington, KY USA; ^4^ Sanders‐Brown Center on Aging, University of Kentucky, Lexington, KY USA; ^5^ University of Florida, Gainesville, FL USA; ^6^ University of Kentucky Sanders‐Brown Center on Aging, Lexington, KY USA

## Abstract

**Background:**

Some types of cancer have been associated with reduced risk of clinical dementia diagnosis. Whether cancer history may be associated with neuropathological features of neurodegeneration or cerebrovascular disease is not well understood. We investigated the relation between cancer diagnosis and brain pathology in a sample of community‐based research volunteers enrolled in an Alzheimer’s Disease Research Center (ADRC) cohort.

**Method:**

Data from autopsied participants from the University of Kentucky ADRC were linked to the population‐based Kentucky Cancer Registery, which is a CDC Surveillance, Epidemiology, and End‐Results (SEER) program. We examined the relationship between cancer diagnosis, clinical dementia diagnosis, Mini‐Mental State Examination scores and neuropathological features using inverse probability weighting to address bias due to confounding and missing data. To address bias due to inclusion of participants with dementia at cohort baseline, we repeated all analyses restricted to the participants who were cognitively normal at baseline.

**Result:**

Included participants (n = 785) had a mean ± standard deviation age of death of 83.8 ± 8.6 years; 60.1% were female. Cancer diagnosis was determined in 190 (24.2%) participants. Participants with cancer diagnosis had lower odds of mild cognitive impairment or dementia, and higher cognitive test scores (e.g. Mini‐Mental State Examination scores evaluated 6 and ≤2 years antemortem, P < 0.001 for both comparisons). Cancer diagnosis also associated with lower odds of higher Braak neurofibrillary tangle stages (III/IV) or (V/VI), moderate/frequent neuritic plaques, moderate/frequent diffuse plaques and moderate/severe cerebral amyloid angiopathy (all P < 0.05). By contrast, TDP‐43, α‐synuclein and cerebrovascular pathologies were not associated with cancer diagnosis. Cancer diagnosis was associated with a lower burden of Alzheimer’s disease pathology and less cognitive impairment.

**Conclusion:**

This study provides evidence that the inverse association between cancer and Alzheimer’s disease may not be fully explained by selection and survival bias. Ongoing research by our team is investigating the association between genetic burden of cancer risk and neuropathological features.

